# A bird vocalisation dataset of birds in Uganda for automated bio-acoustic monitoring and analysis

**DOI:** 10.1016/j.dib.2024.110433

**Published:** 2024-04-16

**Authors:** Mark Abraham Magumba, Tom Denton, Mutesasira Bashir

**Affiliations:** a Makerere University, Uganda, College of Computing and Information Sciences, P.O.Box 7062, Kampala, Uganda; b Google Inc, Uganda

**Keywords:** Bio acoustics, Ornithology, Machine learning, Deep learning, Artificial intelligence

## Abstract

This paper is a description of a bird vocalisation dataset containing electronic recordings of birds in Uganda. The data was collected from 7 locations namely Bwindi impenetrable forest, Kibale forest national park, Matheniko game reserve, Moroto district, Kidepo National Park, Lake Mburo National Park and Murchison Falls National Park. The data was collected between May and June 2023. All together there are 570 recordings from 212 unique species amounting to more than 4 hours of audio. This represents a significant addition to the publicly available electronically recorded vocalisations for birds in Africa. The research was funded by Google Africa Research Collabs for the project entitled, “BASIS: Broad Avian Species Surveillance with Intelligent Sensing”

Specifications Table

**Table:** 

Subject	Bioacoustics
Specific subject area	Automated Bioacoustics
Type of data	Raw
Data collection	The data were collected by different research assistants in the field using an iPhone X and two Zoom Handy H1n Recorders. The birds are identified by ear and by sight. This process is entirely reliant on the skill of the recordist. The data were recorded in a variety of native formats such as .m4a, .mov and .wav.
Data source location	The data was collected from 7 locations namely Bwindi impenetrable forest, Kibale forest national park, Matheniko game reserve, Moroto district, Kidepo National Park, Lake Mburo National Park and Murchison Falls National Park.
Data accessibility	**The data is available at this repository**: Repository name: Mendeley data Data identification number: 10.17632/98hkt6n59j.4 Direct URL to data: https://data.mendeley.com/datasets/98hkt6n59j/4 Instructions for accessing these data: Download and unarchive the zip file

## 1 Value of the Data

• The bird recordings may be used to augment existing data to create or evaluate machine learning models for a variety of applications like automatic bird recognition, creating and improving bird sound embeddings with architectures like Vector Quantized Variational AutoEncoders
• These bird vocalisations may be used for studies in ornithology such as studies in bird behaviour by analysing bird song structure as birds primarily communicate using audio and visual signals
• The recordings can be used evidence of the presence of the detected bird species in the surveyed areas for conservation and environmental monitoring purposes

## 2 Background

Birds are an essential part of any terrestrial ecosystem and perform important tasks like pollination and seed dispersal. Unfortunately, as many as half of the approximately 11,000 bird species in the world are in decline and at least an eighth are endangered [1]. In the specific case of Africa and Uganda, these same statistics hold for instance the latest national red list for Uganda from 2016 considered 156 bird species and found that of the 124 species that weren't data deficient only 19 species weren't threatened in some way [2]. Therefore, there is an obvious need to ramp up conservation efforts. Furthermore, different bird species are finely tuned for different habitats making their numbers a reliable indicator of the health of the ecosystem [3], [4], [5].

However, monitoring bird populations is a difficult task that relies on human skill to identify different bird species. In Africa alone there are about 2477 bird species of which nearly half are represented in Uganda [6] so this is an incredibly difficult and expensive task to perform regularly. Emerging machine learning techniques such as deep learning have made it possible to automate certain tasks like visual and audio bird identification but these first require abundant data for analysis but due to limitations like a lack of recording equipment and skilled individuals there are very few available electronic recordings from Africa. For instance, on the largest such electronic database xeno-canto.org as of February 2024 there were only 3660 recordings from Uganda vs 11081 recordings from Canada and 69123 from the United States. Any machine learning models created from this data would be more accurate on North American species than African species. Therefore, there is a need to collect more African data to improve the performance of these models for African species.

## 3 Data Description

**Fig. 1:**
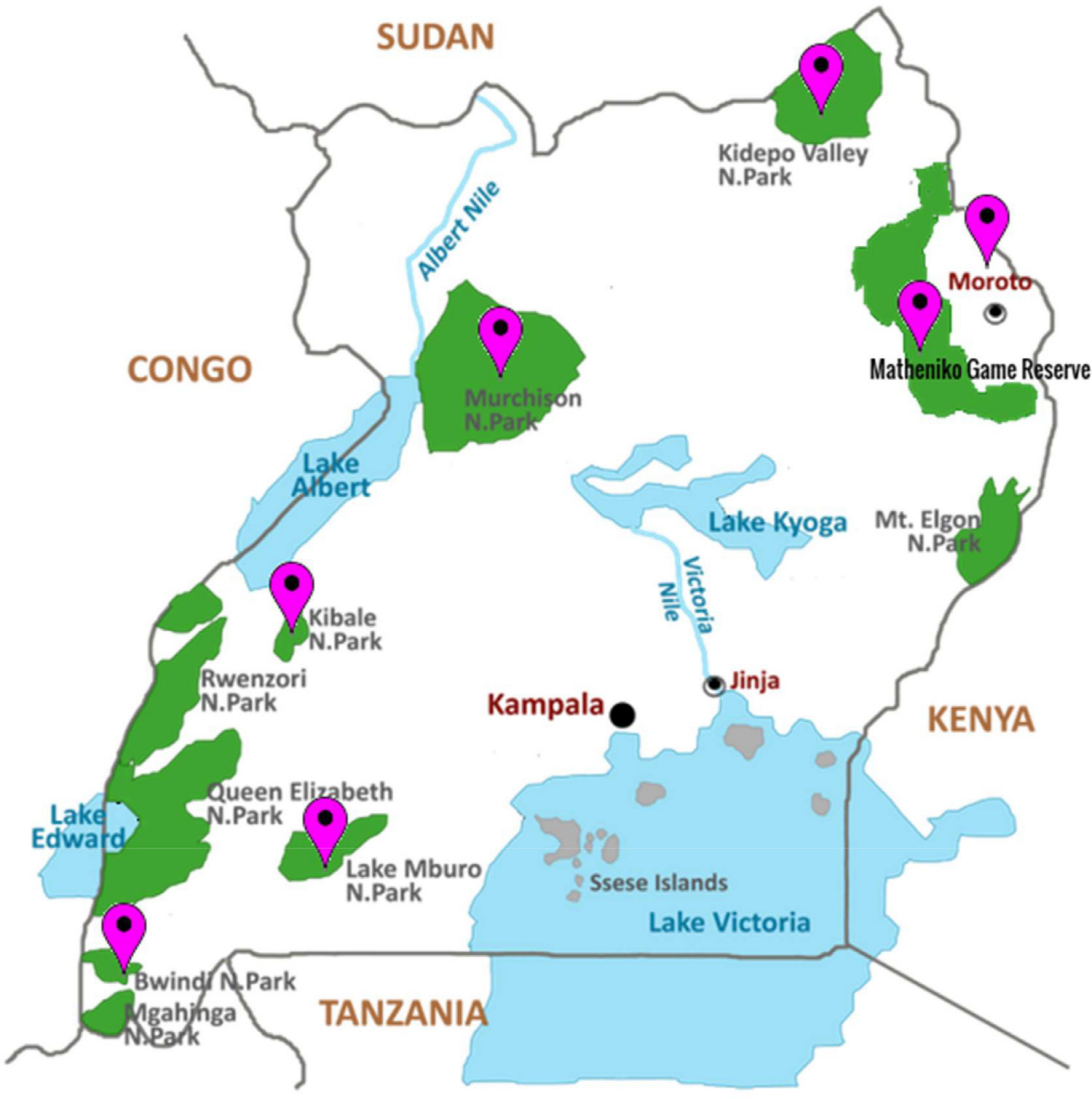
Map showing locations where data collection took place

This data contains 570 files in 8 folders comprising recordings from 7 different locations in Uganda. The recordings are labelled manually by the recordists who rely on their experience to identify the birds. The details of the files are summarised in table 1 below: The sample rate refers to the average number of samples obtained in a second, the higher the sample rate the higher the sound quality. All our files are sampled at 44.1 kHz. The bit depth refers to the amplitude resolution and the higher the bit depth the higher the sound quality. The channels refer to the number of sound channels. Mono implies one microphone (channel) was used for recording and stereo implies two microphones with a left and right input. Stereo sound is more immersive. The last column indicates the number of unique species encountered per location. In interpreting this column, it must be understood that the numbers are not representative of the relative species diversity. These are merely the species we detected at the given time per our constraints. Additionally, the sum of the values exceeds the total number of species detected because many species like Blue-naped Mousebirds appear in more than one location. Table 2 shows a summary of the most frequently sighted birds and Fig. 1 shows the locations where data was collected.

**Table 1:** Summary of data

Location (habitat type)	Folder	Number of recordings	Device	Encoding/ Compression	Sample Rate	Bit depth	Channels/ Average Duration (secs)	Species Detected
Bwindi Impenetrable forest (Afromontane lowland forest)	Bwindi	14	iPhone X	Advanced Audio Coding (.aac)	44.1 kHz	32 bit float	Mono (53.5s)	12
Kibale Forest National Park (Tropical forest to woodland and savannah)	Kibale	79	Zoom Handy Recorder H1n	Wave Audio File Format (.wav)	44.1 KHz	32 bit float	Mono (60 s)	75
Kidepo National Park (Savannah grassland)	Kidepo	105	Zoom Handy Recorder H1n	Wave Audio File Format (.wav)	44.1 KHz	32 bit float	Stereo (17.86s)	32
Lake Mburo National Park (shrublands, grasslands, seasonal and permanent swamps, rocky outcrops and thickets)	Lake Mburo	62	Zoom Handy Recorder H1n	Wave Audio File Format (.wav)	44.1 KHz	32 bit float	Stereo (24.48s)	49
Matheniko Game reserve (Semi-dessert / Shrubland)	Matheniko	102	Zoom Handy Recorder H1n	Wave Audio File Format (.wav)	44.1 KHz	32 bit float	Stereo (17.18s)	30
Moroto (Savannah grassland)	Moroto	10	Zoom Handy Recorder H1n (for .WAV files) and IPhone X for .mov and .m4a files	Wave Audio File Format (.wav), .mov, .m4a	44.1 KHz	32 bit float	Mono (5.88s)	39
	Moroto 2	96	Zoom Handy Recorder H1n	Wave Audio File Format (.wav)	44.1 KHz	32 bit float	Stereo (20.49s)	
Murchison Falls National Park (Grasslands, wooded savannah, tropical forests, wetlands, and open water)	Murchison	102	Zoom Handy Recorder H1n	Wave Audio File Format (.wav)	44.1 KHz	32 bit float	Stereo (18.93s)	39

**Table 2:** Most frequently sighted species.

Species Name	Number of recordings	Location of Sighting
Red-eyed dove	11	Lake Mburo National Park/ KIbale Forest National Park/ Matheniko Game Reserve
Crested francolin	11	Murchison Falls National Park/ Matheniko Game Reserve/ Moroto
Helmeted guineafowl	9	Murchison Falls National Park/ Matheniko Game Reserve/ Lake Mburo National Park
White-bellied go-away-bird	9	Matheniko game reserve/ Moroto
Green-backed camaroptera	9	Murchison Falls National Park/ Lake Mburo National Park/Moroto
Spot-flanked barbet	9	Murchison Falls National Park/ Lake Mburo National Park
Vitelline masked-weaver	8	Murchison Falls National Park/ Kidepo National Park
White-headed Buffalo-Weaver	8	Kidepo National Park/ Moroto
White-crested Turaco	8	Murchison Falls National Park/ Matheniko/ Moroto

## 4 Experimental Design, Materials and Methods

These are focal recordings meaning the recordists actively sought out birds for the purpose of recording their vocalisations in real time as opposed to passive recordings where recording devices are left in the wild and gathered later. Since the project had the explicit objective of obtaining data to train or evaluate a machine learning model we tried to capture as much variability as possible. First, we tried to capture as many species as possible, therefore we limited the number of multiple recordings for any given species to less than 15. As can be seen in Table 2 above the species with the highest number of recordings have 11 recordings. Secondly, we did not record for long durations and in general recordings are below 25 seconds. The reference model was the one used by Google's perch project. This model requires a minimum audio input of 5 seconds. The exception is in Kibale where the average recording length is 60s. The reason for this is that since we were using a single label for each audio file we needed to obtain more data in Kibale where many different species of birds were occupying the same location and there was difficulty in completely isolating calls. In this case the file label is the species whose call is dominant in the foreground of the recording. Where multiple recordings were possible for a species we took steps to ensure that we try and capture different individuals and different bird song phases. The locations were chosen for their abundance of avifauna and lack of environmental disturbances like engine noises being national parks and game reserves. The recordists followed the established bird trails.

For the iPhone X recordings in the “Moroto” folder there are a variety of file formats and these depended on the apps the recordists chose to employ. The .m4a files were created by the voice memo app, the .mov recordings were created by the camera app in video mode and the .aac files in the “Bwindi” folder were originally captured with the iPhone X and then imported into the Itunes desktop app with the default import settings set to convert to .aac format. Also note that some files may sound sub-par to the human ear but this is inconsequential for most downstream purposes. Recall the typical use case for bioacoustic models is to identify species in the wild with consumer grade devices like mobile phones where perturbations like noise and interference are common. It therefore may be counterproductive to use only high quality, clean sound as the input to these models when in reality such perturbations are the norm. Furthermore, these devices typically have less advanced microphone modules and it's the reason we don't opt for high specification microphones like parabolic microphones.

## Limitations

The main technical limitation of the work is that we were only able to provide file level annotations for the recordings due to time and budget constraints. This means that in recordings where there are multiple species present we provide a single label corresponding to the most distinct vocalisation in the recording. Ideally all species should be labelled with the starting and end times for each vocalisation within the recording. However, the file level labelling is still useful for most downstream purposes like automated species identification.

## Ethics Statement

N/A. This work doesn't involve any animal or human experiments.

## CRediT authorship contribution statement

**Mark Abraham Magumba:** Project administration, Conceptualization. **Tom Denton:** Project administration. **Mutesasira Bashir:** Project administration.

## Data Availability

Ugandan Bird Vocalizations (Original data) (Mendeley Data).
